# Why do you think you still have pain? Individuals’ beliefs on the biopsychosocial factors that contribute to their chronic musculoskeletal pain: a qualitative exploration

**DOI:** 10.1186/s12891-025-09243-1

**Published:** 2025-12-24

**Authors:** Michael Dunn, Alison B. Rushton, Nicola R. Heneghan, Andrew Soundy

**Affiliations:** 1 University of Birmingham, Centre of Precision Rehabilitation for Spinal Pain, School of Sport and Exercise and Rehabilitation Sciences, Edgbaston, Birmingham, B15 2TT UK; 2 City St. George’s University of London, School of Health and Medical Sciences, Cranmer Terrace, London, SW17 0RE UK; 3 Faculty of Health Sciences, Western University, School of Physical Therapy, Richmond Street, London, ON N6A 3K7 Canada

**Keywords:** Interpretative phenomenological analysis, Chronic musculoskeletal pain, Qualitative, Beliefs

## Abstract

**Background:**

Chronic musculoskeletal pain (CMP) is complex with many biopsychosocial factors that contribute to its development. Existing research has established individuals’ beliefs on the biological factors that contribute to CMP, but not psychosocial factors. The aim of this study was to determine individuals’ beliefs on the biopsychosocial factors that contribute to their CMP, with specific focus on beliefs about psychosocial contributors.

**Methods:**

A preliminary exploration using interpretative phenomenological analysis methods is reported according to the Consolidated Criteria for Reporting Qualitative Research. Adults with CMP were recruited from the general public. Four stages of data analysis were undertaken to identify superordinate and subthemes.

**Results:**

In-depth analysis of *n =* 6 participants’ interviews identified six superordinate themes. Participants with higher disability from their CMP described psychological factors including negative psychological experiences (distress, loss of self-identity, negative thoughts/emotions, and stress), dissatisfaction with healthcare (let down, fobbed off, lack of empathy, lack of trust), and maladaptive coping strategies (catastrophisation, avoidance, external locus of control), and did not believe these contributed to their CMP. Participants with lower disability from their CMP described positive psychological experiences (solution focussed coping, positive attitudes) and believed these contributed to their CMP by reducing its severity. Participants described social factors including historical activities (work, sport, exercise) and believed these contributed to CMP via the perceived impact of activity causing musculoskeletal structural degeneration or injury. Participants believed biological factors such as structural changes (injury, degeneration) were the main cause of the development and persistence of their CMP.

**Conclusion:**

Individuals with higher disability and negative psychological experiences did not believe these were contributory to their CMP, while those with lower disability and positive psychological experiences believed these do contribute to CMP by reducing its severity. Biological factors such as musculoskeletal structural changes were considered the main cause of CMP. Social factors were believed to contribute to CMP through the perceived impact of physical activity on structural changes. Beliefs are not in keeping with contemporary understandings of CMP which may limit engagement with interventions such as exercise or psychosocial therapies.

**Supplementary Information:**

The online version contains supplementary material available at 10.1186/s12891-025-09243-1.

## Background

Chronic musculoskeletal pain (CMP) is the greatest cause of disability worldwide affecting approximately 43% of adults in the United Kingdom [1]. This causes great economic burden with CMP being the most common cause of sickness absence from work (after common minor illnesses) [2] and only 41% of the working age population with CMP in work [3]. The personal burden is also great with many individuals experiencing moderate to severe disability [1], poorer quality of life [4], and higher prevalence of co-morbidities [5, 6]. Despite the continued advancement of healthcare services, the prevalence of CMP is rising [7] suggesting a need to refine management of CMP.

While the biomedical model has historically dominated understandings of CMP, contemporary theories emphasise a biopsychosocial framework which recognises the complex interplay of physical, psychological and social influences on the development and persistence of pain [8, 9]. This challenges traditional understandings of pain, as it is not possible to explain the mechanisms of how psychological and social factors influence pain through purely biomedical explanations orientated around tissue damage. As such, more nuanced understandings of CMP have emerged. These are predominantly orientated around altered immune and nervous system functions and genetic predispositions (reference) that, when combined with MSK tissue damage, much more plausibly account for the impact of psychological and social factors associated with development of CMP [10]. Particularly, a shift toward increased corticolimbic system activity, a region of the brain responsible for emotional context, stress response movement behaviours, decision making and memory, is prevalent in both CMP and psychological disorders, and therefore may offer insight into the possible mechanisms for psychosocial factors to influence pain [11–13]. However, the extent to which individuals with CMP adopt this multifactorial understanding remains unclear.

Experiences inform beliefs and therefore if individuals’ perceptions of CMP are based on past experiences of acute musculoskeletal (MSK) pain whereby pain and dysfunction resolved with healing, they may not have considered other biopsychosocial factors beyond injured MSK structures. This is important because beliefs about the causes of CMP shape attitudes and approaches on how to manage it [14]. This is demonstrated by research that links beliefs of damaged MSK structures with limiting engagement in physical activity due to the perceived threat of causing harm [15], and limiting engagement with psychosocial therapies because they are considered irrelevant [16]. Further to this, there is evidence to support that beliefs can affect pain. For example, catastrophised perceptions of structural damage or excessively negative expectations are both associated with increased prevalence and severity of CMP [17, 18]. This suggests a complex interplay between beliefs and CMP; not only can beliefs make CMP more likely and more severe, but beliefs may also inhibit engagement in biopsychosocial interventions [15, 16] that are shown to help [19]. Therefore, we need to understand individuals’ beliefs about CMP.

Qualitative studies afford some insights with three studies having examined beliefs about lower back pain [20–22] and one reporting how young people conceptualise the biology of CMP [23]. These studies identify biologically orientated beliefs, particularly that damaged MSK structures cause CMP, but did not specifically enquire about nor report on beliefs about psychological or social factors. Other qualitative studies specifically look at psychological or social factors and identify experiences of emotional distress, work-related stress, or interpersonal challenges but situate these within coping or meaning-making narratives [24, 25] or frame these findings as consequences of CMP rather than contributors [26, 27]. Importantly, no qualitative studies to date have explicitly asked individuals with CMP if they believe their psychological or social experiences contributed to the development or perpetuation of their CMP. Qualitative research has consistently identified catastrophised beliefs orientated around ongoing structural vulnerability contributing to maladaptive behaviours such as fear avoidance [20–22], which has led to fruitful avenues of research and clinical practice such as pain science education [16, 19]. However, beliefs on psychosocial factors that may contribute to CMP are not well explored. Therefore, a qualitative preliminary exploration study is needed which accounts for the whole spectrum of beliefs individuals hold, including psychological and social factors, as well as biological factors. This will highlight areas of focus for future research to address any unhelpful beliefs, identify possible issues, and inform the overall scope of research on this topic.

## Methods

This paper presents part of the findings of a broader study exploring the beliefs of individuals living with CMP. The published study protocol [28] outlines two main aims; to understand what individuals’ beliefs are about the biopsychosocial factors that contribute to their CMP, and to understand how individuals develop their beliefs. Data analysis revealed conceptually distinct findings between these two research aims. Therefore, the decision was made to present the findings across two research papers to preserve the nuance, depth and analytic complexity of the findings for each aim.

### Aims and objectives

To understand individuals’ beliefs on the biological, psychological and social factors that contributed to the development and persistence of their CMP.

### Design and theoretical framework

This qualitative study was designed and reported using the Consolidated Criteria for Reporting Qualitative Research (COREQ) [29] (Additional File 1 – Completed COREQ) with the study protocol published a priori [28].

A preliminary exploratory study is a small-scale, initial investigation conducted when little is known about a topic, with a key aim being to clarify the nature of the phenomenon to help shape future research [30]. This is particularly appropriate for gaining familiarity with a poorly understood problem such as beliefs about psychosocial contributors to CMP, conducted on a small scale with flexible designs.

The research methods for this preliminary exploration are underpinned by Interpretive Phenomenological Analysis (IPA) and data was collected using semi-structured interviews. IPA is an appropriate methodology for studying beliefs about CMP for several reasons. Firstly, IPA is grounded in phenomenology which emphasises how individuals make sense of their personal, lived experiences [31]; essential for understanding beliefs on CMP. IPA also takes an idiographic approach with a focus on in-depth experiences of a small number of participants which allows for detailed, nuanced understandings [31] which is especially important in CMP where beliefs about contributors may be deeply personal and varied. Furthermore, the double hermeneutic process within IPA, whereby the researcher observes and interprets the participants sense-making of their own experiences [32], is crucial in pain research as people with CMP may develop theories about why they experience pain which may influence other relevant factors such as behaviours of daily living, coping strategies, and engagement with healthcare; the double hermeneutic is well suited to explore this interplay which may not be apparent to the participant. Additionally, understanding these beliefs through the double hermeneutic can reveal the detail required to enable clinicians to address beliefs more effectively in clinical practice. CMP is a complex phenomenon involving physical, psychological and social dimensions; IPA accommodates this complexity by allowing rich, multi-layered accounts of pain and its impact on identity, relationships and daily life, which is needed for this study.

This research is situated within a constructivist research paradigm, a branch of interpretivism which places added emphasis on how individuals construct meaning through experience, interaction and dialogue within their own social and cultural contexts, with knowledge co-constructed through the interpretative lens of the researcher [33, 34].

### Sampling

A purposive sample was used with the main criteria being presence of CMP without a clear cause. A sample size of 6–12 was planned in keeping with IPA methods to maintain depth of analysis [31]. Due to the preliminary exploration nature of the study, a maximum variation sample was intended to gain understandings of a broad range of beliefs of people from a rich variety of backgrounds which then may inform areas of focus for future research. This was achieved in terms of socioeconomics, but not ethnic or younger adults. Efforts were made to reach these groups by expanding methods of recruitment by circulating the study advert to these groups through social media (Facebook, Twitter/X), but this was not effective. These recruitment difficulties are dissected within the discussion, with recommendations made to overcome these barriers within future research.

Smaller sample sizes are common in qualitative research which values depth over breadth, and specifically, a sample size of 4–10 is recommended for IPA research [35–37]. Larger sample sizes are appropriate within quantitative research aiming for statistical generalisability; this is not the aim for qualitative research [38] which seeks to achieve naturalistic generalisability where the results have meaning if the “ring true” for the intended population [39].

#### Inclusion criteria

Any adult (> 18 years) with musculoskeletal pain which has been present for at least 3 months.

#### Exclusion criteria

Individuals were excluded if they were unable to communicate verbally and fluently in English, have a high risk or evidence of poor tissue healing (e.g., immunosuppression), have injuries where tissue healing may not be complete at 3 months (e.g., fractures), have pain which is non-musculoskeletal related chronic pain (e.g., cancer) or if they have CMP in the presence of systemic inflammatory conditions (e.g., spondyloarthropathy, rheumatoid arthritis).

### Recruitment

Members of the public were made aware of the study through advertisement. This included advertising to a patient and public mailing list at the University of Birmingham, and specialist interest groups on social media (e.g., Pain UK on Facebook). The study advert was also circulated on social media (Twitter/X). Potential participants who contacted the lead researcher (MD) via email were provided with a study Participant Information Sheet and were screened for eligibility with a telephone conversation.

### Semi-structured interviews

One semi-structured interview was conducted by the lead researcher (MD). The lead researcher is male, was experienced in research, Masters level trained in qualitative methods, a senior practicing musculoskeletal physiotherapist with 10 + years’ experience in the NHS, and a Masters of Research student when the interviews were conducted. All participants were aware of these details and the reasons for conducting the research. Interviews were all conducted within 3 weeks of obtaining informed consent and were conducted remotely using a secure online video platform (Zoom). The interview schedule was informed by the biopsychosocial model of health, an extensive umbrella review of the factors associated with development of CMP [10], the expertise of the authors (MD, AR, AS & NH) and input from patients and public through a meeting with a Patient and Public Involvement (PPI) group with CMP. The original interview schedule was tested with two pilot interviews which identified some issues with flow and ease of understanding of questions. This was therefore updated since publication in the protocol [28] prior to use with participants (Additional File 2 – Interview Schedule) without further amendment thereafter. Whilst a key aim of this research was to identify beliefs particularly about psychological and social factors, the interview schedule was designed to ascertain participants honest, uninfluenced beliefs about their CMP, and therefore the interview schedule does provide opportunity to discuss beliefs about any factors that contributed to their CMP, with specific sections on psychological and social factors included. Interviews were audio recorded and transcribed verbatim by the lead researcher. Field notes were taken. No prior relationship existed between participants and the lead researcher.

### Data analysis

**Fig. 1:**
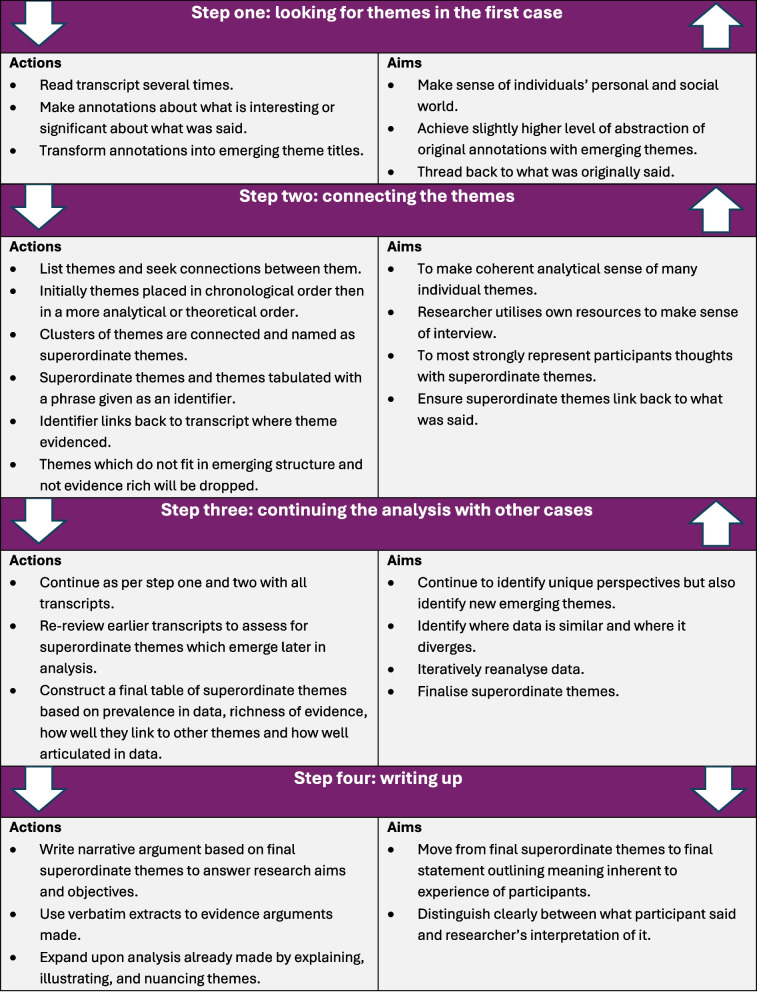
Interpretative Phenomenological Analysis methods based on Smith and Osborn, 2008 [28]

Four steps of analysis were based on Smith and Osborn [31] as published in the study protocol [28] (Fig. 1). ‘Data saturation’ is a concept commonly used in qualitative methods however it was not employed within this study as reaching an endpoint where no new knowledge can be ascertained, despite being a qualitative method, is more coherent with quantitative research philosophies and therefore incongruent with the values and assumptions of the constructivist paradigm within which this research is situated [40]. Analysis was considered to be complete upon achieving understanding and coherence whilst preserving nuance [32]. MD read each transcript several times and applied coding to identify preliminary themes. MD grouped themes under superordinate themes illustrated with direct quotations. Themes were then critically discussed and refined iteratively amongst all co-authors (MD, AS, AR & NH). Themes were inductively derived from the data. Themes and codes were managed using Microsoft Excel. All supporting quotations used for analysis are provided in Additional file 3.

### Strategies to ensure trustworthiness

A reflexive account of the dynamic research process throughout this study has been provided within this article [32], including acknowledgement of any limitations; the hermeneutic circle has been considered throughout interpretation for analysis [32]; ‘member checking’ has been performed [41]; and lastly, validation checks of interpretation of the lead researcher (MD), including scrutinization of any preconceptions or biases, was considered through discussion and feedback with co-authors (NH, AS & AR) [41].

### Ethical considerations

Informed consent was obtained from all participants [28]. Ethical approval was acquired from the Research Ethics Office at the University of Birmingham, United Kingdom (reference ERN_21-0813) and conducted in accordance with the declaration of Helsinki [42]. Anonymity has been maintained; the names of participants used in this article are pseudonyms.

### Patient and public involvement

PPI has been integral to the development of this research study. The lead researcher conducted an interactive discussion with members of the public with CMP from the Centre for Precision Rehabilitation for Spinal Pain PPI Group at the University of Birmingham as detailed within the published protocol [28].

## Results

### Participants

Ten potentially eligible participants were assessed for eligibility. Eight were eligible, *n =* 2 did not respond to further email communication following the screening phone call (no reasons provided) and *n =* 6 completed informed consent and participation. All interviews lasted 50–70 min and were conducted with interviewees at their own home with no one else present. See Table 1 for characteristics of participants, and Table 2 for a description of each participant’s CMP, vocation, and hobbies. CMP presentations across participants are similar with all six participants having multi-site CMP, including five participants with chronic lower back pain and four with chronic knee pain.

**Table 1:** Characteristics of participants (obtained from participants)

		*n*
Age, years	40–49	1
	50–59	3
	70–79	1
	80–89	1
Sex	Male	2
	Female	4
Current work status	Retired	1
	Unable to work due to CMP	1
	Employed full time	4
Highest educational award (or equivalent)	College/sixth form	2
	University undergraduate degree/qualification	2
	Doctoral	2
Current avg. household income	£0–20 k	1
	£20–40 k	2
	£60–80 k	2
	£200 k +	1
Ethnicity	White British	5
	White Jewish	1

**Table 2:** Details of participants CMP, vocation, hobbies and activities

“Charlotte”	Chronic lower back and bilateral knee pain, starting 20 years ago. Retired secondary school science teacher. Used to enjoy long walks, now does shorter walks, swims and does embroidery
“Tony”	Chronic lower back, neck, bilateral ankle, wrist, and knee pain, fibromyalgia, starting 20 years ago. Ex British Armed Forces, then IT engineer, now medically retired due to CMP; attends Help for Heroes meetings and veterans club, but spends time at home mostly due to pain
“Catherine”	Chronic lower back and leg pain, bilateral fingers, hand and foot pain, starting 30 years ago. Works as a doctor (previously GP, now occupational medicine). Goes to the gym, enjoys arts and crafts
“Bethany”	Chronic lower back, neck, hip, bilateral knee, bilateral hand pain, fibromyalgia, starting 22 years ago. Previously worked as a community carer, now a school trauma counsellor. Previously very active with “mum stuff” (walking dogs, park with kids, playing, walking, household tasks) but now can’t do this, swims occasionally if able, but spends time at home mostly due to pain
“Hannah”	Chronic neck, bilateral shoulder and arm pain, fibromyalgia, starting 7 years ago. Previously worked as a teacher (describes as “burnt out teacher”), now does desk-based work in nature conservation. Used to cycle, run and swim, now does yoga, walks dog, does arts and crafts, continues to swim
“Edward”	Chronic lower back, bilateral knee, wrist, finger and toe pain, starting 20 years ago. Previously a teacher and teacher trainer, now semi-retired teaching consultant. Does long walks, volunteers in teaching associations and public research involvement

### Individuals’ beliefs on the factors that contribute to their CMP

#### Psychological factors

There was disagreement across participants for beliefs on the influence of psychological factors on CMP and therefore considerations were made for potential explanations for this within the analysis. Two participants (Tony, Bethany) described a higher impact of CMP on their lives (HCMP) having significantly reduced or modified their activities including stopping work; two participants (Catherine, Hannah) described a moderate impact of CMP (MCMP) having changed some activities; and two participants described little impact from their CMP (LCMP) (Charlotte, Edward) with activities relatively unaffected. Psychological themes are linked to these participants throughout this section based on this observation. See Additional file 4 for the psychological factors coding tree.

##### Superordinate theme 1: negative psychological experiences do not contribute to CMP (Tony, Catherine, Bethany)

Participants with HCMP and one participant with MCMP described negative psychological experiences including psychological distress, loss of self-identity, stress, and negative thoughts and emotions, in relation to their CMP, but did not believe that these experiences contributed to their CMP.

- *Sub-theme 1a: psychological distress (Tony, Bethany).* Participants spoke of psychologically distressing experiences including loss of physical function, bullying, and feeling invisible. Tony stated “So there are things that have from my mental side probably affected me. Things like kangaroo courts and battery barbs, a lot of bullying”.
- *Sub-theme 1b: loss of self-identity (Tony, Bethany).* Participants compared against their former selves, before CMP. Bethany stated “this isn't who I am inside. Inside I'm an able-bodied 17-year-old”. Tony described it as “it's like you lose a part of yourself, I think… you like, when you're doing stuff, like, say you're on a run or a tab, or whatever, you know, and you’ve fallen behind because of that injury, you know, it affects you, and mentally as well that, you know, you know you shouldn't be there but you are there, and you know, that injury has made you be there, say, in the back of the pile or whatever”.
- *Sub-theme 1c: stress (Catherine, Hannah).* Participants described periods of significant stress which co-existed with CMP: “I had a stressful, very stressful, time in my late thirties which led to the divorce. And then I was also a GP partner by that point, so I did have a lot of stresses” (Catherine).
- *Sub-theme 1d: negative thoughts and emotions (Tony, Catherine, Bethany).* Participants described negative emotions including stress, feeling low, depressed, frustrated, angry, lonely, and low self-worth. “There’s been days where I think people would be better off sometimes. I’ve told my husband that if he wants to leave because he don't want left with, not a vegetable, but a less able body, because I'm only going to get worse as years go by, then to go now. So I push people away. I go to bed sometimes, and I just don't want to wake up” (Bethany).

Participants were asked if they believed these negative psychological experiences may have contributed to their CMP. Catherine, after acknowledging significant stress that led to a divorce, went on to say “But they didn't, yeah, they didn't impact on, I don't think they impacted on my pain”; Tony said “I don't think they knock on as much. They probably do affect the mental side more than the physical. Definitely, yes, but yeah, not the other way around”; and Bethany said “I just think it's the compression. I think it is down to the nerves, and the biology of it all. I don't think it's down, I don't think it's a psychological issue”. Contrary to this, Hannah (MCMP) acknowledges the impact of stress on her CMP, and Charlotte and Edward (LCMP) did not describe any negative psychological experiences.

##### Superordinate theme 2: unsatisfactory healthcare contributes to CMP (HCMP)

This theme is based on two participants with HCMP who identified dissatisfaction with their healthcare as being contributory. Healthcare experiences were not part of the interview schedule, therefore participants volunteered these beliefs without being directly asked, suggesting these were meaningful beliefs.

- *Sub-theme 2a: let down by healthcare services (Tony, Bethany).* Participants provided frequent experiences of feeling let down by healthcare services due to being denied access to further investigations, referrals to specialists, or medications: “It's like you've got to prove that you're in this pain constantly, that you've got to somehow show them their job… And I feel let down, if I'm being honest. That I've just been thrown in the sea to survive. I'll throw you a raft every now and then, but it's got a bit of a puncture, so you have to keep asking us for some more air. So that when I have a big flare up, it's like trying to get crack cocaine off the Queen or something [getting painkillers from the GP]” (Bethany).
- *Sub-theme 2b: fobbed off (Tony, Bethany).* Referrals to healthcare services which are not valued by participants were perceived as a way of getting rid of them. Talking about a pain management programme, Bethany stated “there was something missing in me, and it was like mourning my old life. The old Bethany. But they don't teach you that in these [pain management] courses, and I just felt like it was just, just a way to get rid of you”.
- *Sub-theme 2c: lack of empathy (Tony, Bethany).* These frequent experiences of feeling let down or fobbed off could be perceived as a lack of empathy from healthcare professionals: “Sometimes, people didn't have empathy when I was going through it, and they didn't believe what you had, and it was a struggle to get that help. Like, you know, I want a referral to see a specialist and the doctors say ‘no you can't have it’” (Tony).
- *Sub-theme 2d: lack of trust in healthcare professionals (Tony, Bethany).* These experiences form the basis for a lack of trust in healthcare professionals with participants challenging competence and agendas: “And this is why you get crap doctors and crap carers and stuff like that, because, they don't understand, and they’re there for money, if anything. You need to put your heart in a job before you look at the wages” (Bethany).
- *Sub-theme 2e: more peer support options would help (Bethany, Hannah).* Two participants independently identified that having peer support options from within healthcare would have helped manage their CMP: “So, I think if my GP had said to me ‘I think you have fibromyalgia, here's a support group you can go and talk to locally’, or, you know, ‘here's someone who is a coach and can help coach you through, you know, what you're experiencing’. I think that would have been helpful” (Hannah).

##### Superordinate theme 3: maladaptive coping strategies do not contribute to CMP (Tony, Catherine, Bethany, Hannah)

All participants with HCMP and MCMP spoke about their thoughts, attitudes and behaviours toward managing CMP which were in keeping with known ‘maladaptive coping strategies’ [43]. This included catastrophisation, avoidance, and external locus of control.

- *Sub-theme 3a: catastrophisation (Charlotte, Tony, Catherine, Bethany, Hannah).* Most participants exhibited elements of catastrophisation or fear. This included negative self-statements, expectations of negative outcomes, or excessively negative perceptions or descriptions of structural changes. Bethany made self-statements such as “I've just got crap bones”, and had an excessively negative perception of her spine: “the discs in between obviously degenerate, so they’re crumbling”.
- *Sub-theme 3b: avoidance (Catherine, Bethany, Hannah)*. Participants described experiences such as reducing mobility, increasing use of support aids, increased rest, avoiding tasks, changing career, or stopping work altogether. “So as soon as I discovered that I got slipped discs in 2016, I stopped doing some activities, I stopped riding my bike, I used to run a bit, I was never a natural runner, but I stopped doing that” (Hannah).
- *Sub-theme 3c: external locus of control (Tony, Bethany).* Participants with HCMP demonstrated an external locus of control exampled by Bethany’s reenactment of seeking pain medications from the GP: ““Well, why do you need it?” it's like, “because I'm having a flare up”, “but why don't you try this instead?”, it's like “because that doesn't work for me”” (Bethany).

When asked if these maladaptive coping strategies affected their CMP, Catherine, Hannah (MCMP) and Bethany (HCMP) all agreed that their approaches did not contribute to their CMP. Catherine, when asked if stopping or avoiding activities may have made CMP worse stated: “The things that we've mentioned? No, no, it helped. They all helped”. Tony (HCMP) acknowledged that avoidance may have made CMP worse, and Edward and Charlotte (LCMP) did not describe maladaptive coping strategies.

##### Superordinate theme 4: positive coping strategies improve CMP (Charlotte, Catherine, Hannah, Edward)

Participants with LCMP and MCMP described thoughts, beliefs and behaviours in keeping with positive coping strategies [43] and believed these improved their CMP by reducing it or preventing it from worsening.

- *Sub-theme 4a: solution focussed coping (Hannah, Charlotte, Edward).* Solution focussed coping is when one manages their CMP by changing the situational stressor or changing oneself (e.g., reappraising the situation, information seeking) [43]. Hannah particularly embraced this: “the GP said to me “I think you've got fibromyalgia”. I mean it's never been a formal diagnosis; for me, I don't really need one. I'm happy to work with that and manage it from what I've read about it since, it seems to make sense to me, but having something that I can then go research and work out how to work with helped me, because I like to know about things”.
- *Sub-theme 4b: positive attitudes (Edward, Hannah, Charlotte).* Participants spoke about their positive attitudes. This includes experiences of self-reassurance, rationalising, perseverance of activity and taking ownership/control. Edward particularly demonstrated this approach: “Going into a hospital, probably, that event probably did frighten me a bit, but it didn't frighten me in the sense of ‘my God, this is the end of my life’, or ‘I've been doing something totally wrong and I must change everything’. My main concern was to get back to work as quickly as possible, which I suppose again, is a positive thought rather than a negative one”.
- *Sub-theme 4c: exercise (Edward, Charlotte, Hannah, Catherine, Tony).* Most participants agreed that exercise was helpful for CMP. Edward, who generally does not hold catastrophised beliefs, put it this way: “Some people say you should rest but I don't believe that. I think you have to continue to use ligaments or joints that are not working properly”. Tony and Bethany (HCMP) also agreed exercise was helpful, but had difficulty engaging with exercise due to their CMP which instead led to avoidance: “Oh, I'll just avoid because it’s too much effort, and hurting. I usually… because I'm in the water and, this is why I stopped my hydrotherapy is, thinking I'm invincible, and then I suffer for like three days afterwards” (Bethany).

Participants with positive coping strategies believed that their CMP was better because of their approaches. Edward articulated this well for exercise and a positive attitude: “as these [joints] are living things, they presumably have the power to keep themselves repaired as much as possible. So, I believe that usage does continue to help the repair process, and non-usage tends to encourage it not to repair, and therefore to get worse”, “I think a positive attitude is the most important thing; not saying ‘oh, dear, I shall never walk again’ which presumably some people do say”.

#### Social factors

##### Superordinate theme 5: historical activities contribute to CMP

Participants described past experiences including work, exercise and hobbies which they believed contributed to their CMP based on the perceived impact of the activity on structural changes. See Additional file 4 for the social factors coding tree.

- *Sub-theme 5a: work*. Participants identified work as contributory to their CMP and believed this contributed to structural changes. Tony stated “you’re always trying to do 120 percent… so you didn’t get down from a tank, you jump down from a tank… so yeah, all that, yeah, has gone to the back pain. And now I got my MRI was, yeah, the L2, 3, 4, 5, and S1 bulging”. Charlotte believed it was to do with carrying books in her job as a teacher, and has since made this connection to her CMP retrospectively: “it's only when you start to think back and think, why have I got this? What caused this? But you, if you think back, and think oh, maybe it was that?”.
- *Sub-theme 5b: exercise and hobbies.* Participants also make similar connections to previous exercise including walking, sports, training and arts and crafts. Hannah stated “I put it [CMP] down to playing a lot of sport when I was a teenager, I was outside a lot just putting my body through a lot of physical stuff”.

#### Biological factors

##### Superordinate theme 6: biological factors are the main reason for CMP

All participants articulated biological factors they believed contributed to their CMP including structural changes and posture. Participants often based other beliefs, such as psychological or social factors, on their ability to link these back to perceived biological factors; for example, Tony stated “I've definitely got arthritis in both my wrists and that could be related from, I'd say, the IT work and the way, the position the hands are all the time”. This suggests that biological factors were the overarching belief to explain CMP. Furthermore, at the end of the interview participants were asked what their “main” belief was about the cause of their CMP with five participants describing biological factors. See Additional file 4 for the biological factors coding tree.

- *Sub-theme 6a: structural changes.* All participants provided an explanation of structural changes that they believed to be contributory to their CMP. “So, to my knowledge degenerative discs is the discs in between obviously degenerate, so they’re crumbling, and I know that one of my discs are quite low, so it's near bone on bone. So, if I move funny, the pain goes through my whole body, and then I obviously get bulging discs” (Bethany).
- *Sub-theme 6b: posture.* All participants at some stage identified posture as a contributor to their CMP, often alongside other beliefs. Hannah, describing the cause of her “slipped discs” and subsequent CMP as “I think a lot of it was down to stress and poor posture”, while Catherine highlighted “degenerative changes and poor, erm, poor ergonomics” as the main reason for acquiring CMP.

## Discussion

This preliminary exploration study using IPA methods is the first study to explore individuals’ beliefs on the biopsychosocial factors that contribute to their CMP, with specific enquiry and detail on psychosocial beliefs, with new understandings identified about the interplay between beliefs. An in-depth analysis of 6 participants’ beliefs identified six themes and revealed rich understandings of the psychosocial experiences of participants with CMP. Participants who were more disabled by their CMP (changed careers, stopped working altogether, and report substantial impact on interpersonal relationships) described many negative psychological experiences (themes 1, 2 & 3), and did not believe these were contributory to their CMP, but rather a consequence of it. Conversely, participants who were less disabled by their CMP (daily life relatively unaffected) described many positive psychological experiences (theme 4) and believed that these did contribute to their CMP by improving it. Despite the rich level of detail and insight into psychosocial experiences provided, almost all participants believed that structural changes were the main cause of their CMP (theme 6), with social factors such as hobbies, exercise and work (theme 5) all thought to have contributed to development of CMP by means of their causing structural damage or degenerative changes. This was the case even in instances where social factors, such as work or interpersonal relationships, were psychologically distressing (e.g., bullying) or stressful with most participants not believing these were contributory to their CMP. One participant did believe stress was the main cause of her CMP, but even so still described the mechanisms of her CMP purely through biological structural changes; this suggests that even if individuals do believe psychosocial factors contribute to CMP, they may not understand how.

This is consistent with previous research on beliefs about pain which identifies a belief of structural ‘vulnerability’ in the form of injury, damage or dysfunction [21, 23]. Likewise, participants in one of these studies also associated their CMP with past behaviours with the belief that they could injure their back during trivial activities without being aware they were “causing damage” at the time [21]. This is in keeping with the social factors identified in this study (theme 5) whereby historical activities (work, carrying bags and sports) were still thought to have contributed to structural changes, and development of CMP which occurred many years later. Other existing research of young adults with CMP identified additional beliefs such as overactivity of nerves and stress systems [23] which were not identified in our study, likely because their participants had previously undergone pain education. The psychological experiences identified within our study are in keeping with existing research with participants describing such experiences as loss of self-identity, distress and fear [25, 26] and an adversarial experience of healthcare [44] with participants like Bethany and Tony feeling like they must “prove” their CMP, and “fight” for healthcare support. Whereas these studies described these experiences as existing with, or a consequence of CMP, our study specifically establishes that individuals with these experiences may not believe they were contributory to their CMP.

This is important because beliefs heavily grounded in structural changes have been shown to limit engagement in physical activity due to perceived threat of worsening the injury, and limits engagement with psychosocial therapies which may be considered irrelevant [15, 16]. This was the case in our study specifically for individuals with higher disability and more negative psychological experiences i.e., those who could benefit the most from intervention. These individuals did not participate in exercise, whereas all other participants did, and one participant described psychosocial therapies as “a way to get rid of you”. These individuals also had lower household income and educational achievement which raises a concerning question about whether current interventions such as exercise and psychosocial therapies, whilst grounded in research, may be less accessible to those from lower socioeconomic backgrounds, in part, due to their beliefs. Certainly, individuals in our study with lower levels of disability were all from higher socioeconomic backgrounds, and perceived greater impact of psychological factors on CMP and therefore took on positive attitudes and adaptive coping strategies which they believe reduced severity of symptoms (theme 4). This is in keeping with research that links improved understandings of psychosocial factors with improved management of CMP [16, 19] and that the way people make sense of their pain is associated with level of disability [45, 46].

### Implications for clinical practice

This study highlights a number of implications for clinical practice. Firstly, individuals believe that biological factors such as injured or degenerated structures are the main cause of CMP. This is not in keeping with contemporary evidence which situates CMP primarily as a nervous system disorder [47, 48], with wide ranging, non-MSK-structure related biopsychosocial factors that contribute [10]. It is therefore important that clinicians’ possess up to date, evidenced based understandings of CMP, and are practised at explaining this in a range of ways to a variety of patients. However, our study also observed that participants with the highest levels of disability also rejected the belief that their negative psychological experiences contributed to their CMP and felt unable to exercise, highlighting a particular barrier to helpful interventions. These participants were also from lower socioeconomic backgrounds, a known barrier to high-value MSK healthcare in the UK leading to increased opioid dependency [49]. Further consideration is required as to how healthcare systems can better meet facilitate these individuals in modifying their beliefs and engaging in exercise.

### Research limitations and future directions

There are some limitations to this preliminary exploration IPA study. We sought to capture beliefs on CMP from a broad range of individuals to inform avenues for future research on a topic where little is known. We were able to achieve this for in terms of socioeconomics, but notably it was difficult to recruit individuals from ethnic backgrounds with efforts to reach these individuals through social media unsuccessful. Research identifies a number of possible reasons for this including socioeconomic and logistical challenges and mistrust of healthcare/researchers [50]. In light of these issues, it is likely the research PPI mailing list we used for our main source of recruitment was under-representative of ethnic individuals. As such, future research should seek to recruit in settings with higher ethnic diversity, such as certain NHS services or community hubs, and consider the use of personalised recruitment approaches, whilst adhering to non-burdensome data collection methods as used in our study. Three of our six participants were in their 50 s which may have affected results if identified beliefs were particular to this age group. We also did not recruit any individuals under the age of 40 with the most likely reason for this being that the prevalence of CMP increases with age with far fewer young people affected [51]. It is also noteworthy that when younger people do have CMP, this is more likely to be associated with a diagnosis of a mental health condition compared to older individuals [52]. It therefore may be beneficial for future research to focus on younger people separately.

This preliminary exploration IPA study has identified several avenues for future research. Particularly, future research should seek to understand the relationship between level of disability, psychological experiences and beliefs on their contributions to CMP, as our study suggest beliefs differ across these groups. Specifically, a further IPA study focussing on those with high levels of disability is needed to understand psychological experiences and their contribution to CMP in more depth. This is particularly important because of the barriers to engagement with healthcare interventions beliefs may impose, and subsequent healthcare inequalities for these individuals. Further research may also seek to understand the relationship between beliefs and socioeconomics, and also focus on groups of individuals from specific ethnic backgrounds, to better understand the diversity of beliefs that exist.

## Conclusion

Participants with higher disability from their CMP described negative psychological factors and did not believe these contributed to their CMP, while participants with lower disability described positive psychological factors and believed these contributed to their CMP by improving it. Social factors were thought to contribute to CMP, but only through the impact of activities on MSK biological structures. Even with specific discussion around individuals’ psychosocial factors, participants believed that structural changes such as injury or degeneration of MSK structures were the main cause of the development and persistence of their CMP.

## Supplementary Information

Supplementary Material 1.

Supplementary Material 2.

Supplementary Material 3.

Supplementary Material 4.

## Data Availability

All data generated and/or analysed during the current study are included in this published article and its supplementary information files, with the exception of the full interview transcripts which will be stored securely by University of Birmingham for 10 years, then destroyed in accordance with our ethical approval.

## Abbreviations

CMP: Chronic Musculoskeletal Pain
MSK: Musculoskeletal
COREQ: Consolidated Criteria for Reporting Qualitative Research
IPA: Interpretative Phenomenological Analysis
PPI: Patient and Public Involvement
