# Supplementary material for: Why do you think you still have pain? Individuals’ beliefs on the biopsychosocial factors that contribute to their chronic musculoskeletal pain: a qualitative exploration
Source: BMC Musculoskelet Disord. 2025 Dec 24;26:1103. doi: 10.1186/s12891-025-09243-1 (PMC12729344; doi:10.1186/s12891-025-09243-1)
Supplement: Supplementary file 2 — Supplementary Material 2. [file 12891_2025_9243_MOESM2_ESM.docx]

| **Interview Schedule**  ***** Note:** in order to individualise and personalise the semi-structured interview, the interviewer may use the language and terminology used by the interviewee when asking questions. Phrases in the interview schedule where this is considered to be more likely have been **“quotation marked and emboldened”**. | | | |
| --- | --- | --- | --- |
| **Research Aim** | To understand individuals' beliefs and perceptions on the biological, psychological and social factors which contribute to the development and maintenance of chronic musculoskeletal pain. | | |
| **Interview Section** | **Questions/Content** | **Prompts** | **Aims** |
| Ethics Statement | Firstly, thank you for participating in this interview. I would just like to remind you that this interview will be audio recorded to ensure an accurate collection of the insights and information you provide. After the interview I will transcribe our conversation. With your consent, I will share the this with you and may also get in touch in order to clarify the meaning in certain statements you may make. All information you provide will be kept strictly confidential. You are free to stop the interview and the recording at any pointe and end the interview altogether if you wish.  There are no right or wrong answers as I am simply interested in your own experiences, beliefs and thoughts about 1) your pain and, 2) what we already know from research about factors associated with chronic MSK pain.  Before we begin, do you have any further questions? | *Can I confirm that you have read and understand the information sheet?*  *Are you comfortable?*  *Please feel free to stand and move around at any point in order to be comfortable.*  *Please feel free to ask for a break if you need to.*  *Is there anything you don’t understand?* | - To ensure a complete understanding of the purpose of the interview and what is expected of the participant. - Make sure the participant is comfortable and happy to begin. |
| Introductory Questions | 1. Where do you come from? 2. What do you do for a living? 3. What does this involve? 4. How is life at home? 5. Do you have any hobbies, activities, or exercise routines that you do regularly? 6. Is there anything else which regularly occupies your time? | *Whereabouts in the World did you grow up? What kind of area did you live in? Did you enjoy living there?*  *How long have you been in your vocation? Did you do something different before then? Have you changed vocation? Who do you live with? Do you have a partner or spouse at home? Do you have any children? Their age(s)? Is your home life relaxed or quite busy?* | - Help participant relax and feel comfortable. - Build rapport. - To gain insight into the influencers and experiences in the individual’s life which may have helped shape their beliefs and perceptions of their chronic MSK pain. |
| Transition Questions | 1. Throughout our conversation I will refer to your chronic MSK pain in my questions. How would you like me to refer to this? For example, “back pain” or just “pain”, or is there anything else you prefer? 2. How did your **“pain”** begin? 3. How long have you experienced your **“pain”** for? 4. Has your **“pain”** changed over time? 5. When did your **“pain”** become a problem for you? 6. Were there any periods of time when your **“pain”** was better? 7. Why do you believe you developed this **“pain”**? 8. Why do you think you have experienced **“pain”** for as long as you have? 9. Do you think anything more could have been done to help you get better? 10. Do you think you could have done anything differently? | *When you’re talking to someone about your pain, what do you call it?*  *How did it start? How has it changed/evolved? Were there any periods of remission and recurrence?*  *When did it start to cause you to first seek help? When did it first stop you from doing things which were important to you? When did you first start to worry about it?  Do you think you developed this pain due to injury? Due to aging? Due to changing your activities? Due to thoughts and feelings?* | - Begin to guide the interview towards their chronic MSK pain and experiences since onset. |
| The biopsychosocial model in chronic MSK pain | There is lots of research investigating the factors which contribute to people developing chronic MSK pain. The main question being asked is “why do some people get better when others don’t?”. The research aims to understand the differences between those who get better and those who don’t, so that healthcare services can provide better care in helping as many people as possible to get better.  Current research shows there are many different factors which can contribute to the development of chronic MSK pain. These can be placed into three main categories: 1) factors to do with the individual’s body, 2) the way people think and feel, and 3) the activities that people do.  I have some questions for you regarding your beliefs and thoughts as to whether any of these might have contributed to your **“pain”**. If anything doesn’t quite make sense as we go through, please let me know. | *Do you have any questions?*  *Do you understand what I mean by factors to do with your body?*  *Do you understand what I mean by factors to do with your thoughts and feelings?*  *Do you understand what I mean by factors to do with your activities?* | - Inform the participant about the background of the study - Introduce or reaffirm that research shows biological, psychological and social factors can affect chronic MSK pain, and that this is what the questions are based on. |
| Main Questions:  Patient beliefs on biological contributors | This section is about your body. This refers to anything about your body such as physical condition, health, fitness, injuries, age related changes, genetics and any other thing about your body.   1. How was your physical health and well-being in the time leading up to the beginning of your **“pain”**? 2. Do you think anything about your physical health contributed to the start of your **“pain”**? 3. When your **“pain”** started, what do you think happened to your **“area of body”**? 4. Do you think **“these changes”** to the structure of your **“area of body”** have recovered? If so, how long do you think this took? 5. Do you think **“these changes”** have contributed to your **“pain”** not going away? 6. Has your physical health changed since your **“pain”** started? 7. Do you think any of **“these changes”** have contributed to your continued experience of your **“pain”**? 8. Last question for this section, why do you believe these things about your body? Where do these beliefs come from? | *What kind of physical shape were you in? What was your health like at the time?*  *Do you think your shape, fitness or health contribute to your developing chronic musculoskeletal pain?*  *Do you think you injured anything?*  *Do you think these injuries healed? How long did this take?*  *Has your physical health changed since developing musculoskeletal pain?*  *Do you believe these things because someone told you them? Such as a medical professional, friends, family or the internet? Or any other reason why you believe these things?* | - To understand the patients experiences and perceptions of their physical shape and health before and throughout having chronic MSK pain and what they believe regarding the influence this has on their experience of pain. - To understand where these beliefs come from. |
| Main Questions:  Patient beliefs on psychological factors | This section is about your thoughts and the way you’ve been feeling.   1. How were you feeling in the time leading up to the beginning of your **“pain”**? 2. Do you think **“these thoughts and feelings”** contributed to the start of your **“pain”**? 3. How did you feel after your **“pain”** started? 4. What kinds of thoughts were running through your mind? 5. Do you think **“these thoughts and feelings”** contributed to your **“pain”** not going away? 6. Have any of **“these thoughts and feelings”** toward your **“pain”** changed since it started? 7. Do you think any of **“these thoughts and feelings”** have contributed to the continued experience of your **“pain”**? 8. Last question for this section, why do you believe **“these things”** about **“your thoughts and feelings”**? Where do these beliefs come from? | *Were you experiencing any: Increased stress, anxiety, depression, lower mood, concern, worry, fear, unhappiness, frustration, anger, helplessness, loss of confidence, loneliness, isolation.*  *Do you think these feelings could have affected your pain in any way?*  *Do you believe these things because someone told you them? Such as a medical professional, friends, family or the internet? Or any other reason why you believe these things?* | - To understand the patients experiences and perceptions of their psychological health and health before and throughout having chronic MSK pain and what they believe regarding the influence this has on their experience of pain. - To understand where these beliefs come from. |
| Main Questions:  Patient beliefs on social factors | This section is about the things that you do. This includes things such as your vocation, hobbies, exercise, socialising, family responsibilities and any other activities.     1. What things were you doing in your life in the time leading up to the beginning of your **“pain”**? 2. Do you think **“these things”** may have contributed to the start of your **“pain”**? 3. When your **“pain”** first started, did you have to change or modify any of **“these things”**? 4. Do you think any of **“these changes”** to the things you do may have contributed to your **“pain”** not going away? 5. As time has gone on, have you been able to do **“these things”** in the same way you could before the start of your **“pain”**? 6. What kind of things do you do now? 7. Do you think any **“changes”** to **“the things you do”** since the start of your **“pain”** have contributed to your continued experience of your pain? 8. Last question for this section, why do you believe these things about the things you do? Where do these beliefs come from? | *Exercise routines, hobbies, work, family responsibilities, seeing friends, socialising, household responsibilities such as cooking, cleaning or shopping.*  *Any change to any of the above things mentioned.*  *If any of your activities have changed, do you think this has affected your pain?*  *Do you believe these things because someone told you them? Such as a medical professional, friends, family or the internet? Or any other reason why you believe these things?* | - To understand the patients experiences and perceptions of social activities before and throughout having chronic MSK pain and what they believe regarding the influence this has on their experience of pain. - To understand where these beliefs come from. |
| Main questions: Summarising patients main beliefs | These last questions are just to help summarise your main beliefs about the contributors to your chronic MSK pain. Please reflect on your answers from the rest of our conversation to answer these as best as you can.   1. Do you think any of the things we have discussed today have influenced one another? E.g. **“thoughts/feelings”** having any influence on **“things you do”**. 2. What do you think is the main reason you developed your **“pain”** to begin with? 3. What do you think is the main reason your **“pain”** has continued for as long as it has? 4. What do you think needed to happen in order for your **“pain”** to improve? 5. Finally, is there anything you would like to add about your beliefs on the factors that have contributed to your **“pain”**? | *Is there any relationship between your body, your thoughts and feelings and your behaviours? Do you think they affect one another?* | - To understand if patients connect their biological, psychological and social experiences and beliefs. - To distinguish the patients most strongly held beliefs from the discussion. |
| Conclusion | That’s all the questions.  The interview has now finished. Thank you for participating in this study, I really appreciate your time and input. | *Is there anything you would like to ask regarding the analysis of the data or the next steps of the process?* | - Ensure the participant is comfortable with what has been discussed. |
