# Supplementary material for: Why do you think you still have pain? Individuals’ beliefs on the biopsychosocial factors that contribute to their chronic musculoskeletal pain: a qualitative exploration
Source: BMC Musculoskelet Disord. 2025 Dec 24;26:1103. doi: 10.1186/s12891-025-09243-1 (PMC12729344; doi:10.1186/s12891-025-09243-1)
Supplement: Supplementary file 3 — Supplementary Material 3. [file 12891_2025_9243_MOESM3_ESM.docx]

| **Theme** | **Sub-theme** | **Supporting quotations** |
| --- | --- | --- |
| **Biological factors are the main reason for CMP** | Structural changes | **Charlotte** “so you ended up with presumably cartilage on cartilage, but that presumably has worn away. And you've got bone rubbing on bone”  **Tony** “I think it gets worse over time. It's like anything; if you’ve got a cut and you don't put a plaster on it's gonna bleed, you know. And if you leave it over time it can heal, but sometimes it can leave a callous there. So you know, it's not fully healed”  **Tony** “And now I got my MRI, the L 2, 3, 4, 5, and S1 [are] bulging”  **Catherine** “I went to the doctors, and they sent me for an MRI, and that's when they discovered that I’ve got degenerative disc disease in my L4 and L5”  **Bethany** “So then I got diagnosed with the arthritis, and then on my last scan, I can’t remember when it was, a couple of years back, I was I was diagnosed with osteoarthritis in my back, in my spine, so obviously it's gotten worse”  **Bethany** “So, to my knowledge degenerative discs is the discs in between obviously degenerate, so they’re crumbling, and I know that one of my discs are quite low, so it's near bone on bone. So, if I move funny, the pain goes through my whole body, and then I obviously get bulging discs”  **Hannah** “But this pain just didn't go away. And that's when I found out, actually, I’d got some slipped discs. So, the pain I had for that was just horrible… Oh I've got spondylosis as well, I forgot about that”  **Hannah** “They're pressing on something. And so yeah, I couldn't say. But having seen them, and seeing that they're bulging on to something that is triggering pain. Yeah, it must contribute to how I feel”  **Edward** “I did have a slipped disc at one stage, and had to be hospitalised for that” |
|  | Posture | **Tony** “you get your body in a position that’s comfortable, so there’s less stress on the spine, or wherever the pain is”  **Catherine** “Degenerative changes and poor, erm, poor ergonomics, I suppose, you know in my younger age”  **Bethany** “I think it’s more down to my posture now, obviously, I’ve shrunk”  **Hannah** “I think a lot of it was down to stress and poor posture”  **Edward** “I often wonder whether posture has anything to do with it. Poor posture encourages uneven load” |
| **Negative psychological experiences do not contribute to CMP** | Psychological distress | **Tony** “So there are things that have from my mental side probably affected me. Things like kangaroo courts and battery barbs, a lot of bullying, which now, they class as bullying but then it wasn’t”  **Bethany** “The worst one was last, 2019, yeah 2019, my daughter ended up having to help me get off the toilet because, I just, my legs wouldn’t work. And then I crawled back to the chair in the living room where I've got a chair that stands right up, so it stands you up in position, and her and my husband managed to get me onto this chair, and then I ended up like ringing 111 and telling them, and they sent me an ambulance out because of the cauda equina that you can get”  **Bethany** “So it's really hard, and you do feel invisible. They say it’s an invisible illness, and you feel invisible with it” |
|  | Loss of self-identity | **Tony** “it's like you lose a part of yourself, I think. You like, when you're doing stuff like, say you're on a run or a tab, or whatever, you know, and you’ve fallen behind because of that injury, you know, it affects you, and mentally as well that, you know, you know you shouldn't be there but you are there, and you know, that injury has made you be there, say, in the back of the pile or whatever”  **Tony** “So I used to be the life and the soul of the party, and it was like, I went to my mates wedding, and I was sat in the corner not the life and the soul of the party, and my friend comes across and says ‘come on, Tony, what's going on? Where's the old Tony? You know it’s like, yeah, totally new from that point. Other people notice it as well”  **Bethany** “it was the embarrassment, and people think, you know, because you’re fat you’re on a scooter, and, just everything about it, it’s like this isn't who I am inside. Inside I'm an able-bodied 17-year-old, do you know what I mean?”  **Bethany** “there was something missing in me, and it was like mourning my old life. The old Bethany. But they don't teach you that in these [pain management] courses, and I just felt like it was just, just a way to get rid of you” |
|  | Stress | **Catherine** “I had a stressful, very stressful, time in my late thirties which led to the divorce. And then I was also a GP partner by that point, so I did have a lot of stresses. But they didn't, yeah, they didn't impact on, I don't think they impacted on my pain” |
|  | Negative thoughts and emotions | **Tony** “Yeah, I just try and, at the minute it's, I'm just surviving, not living as such I would say”  **Catherine** “I've been frustrated, a bit fed up, and down, particularly with my hands. And when I wasn't sleeping, yeah, I was a bit low, I would say, when it was impacting on my sleep. But frustrated, yeah. And annoyed at times”  **Bethany** “sometimes I just think I'm sick of being in pain, I'm sick of moaning. And if I'm sick of moaning, everybody else must be sick of hearing me moan. And you feel alone.”  **Bethany** “There’s been days where I think people would be better off sometimes. I’ve told my husband that if he wants to leave because he don't want left with, not a vegetable, but a less able body, because I'm only going to get worse as years go by, then to go now. So I push people away. I go to bed sometimes, and I just don't want to wake up” |
| **Maladaptive coping strategies do not contribute to CMP** | Catastrophisation | **Tony** “it's just got the degradation of the discs, and everything else is going down, down, down, probably a lot quicker than it normally would do because of all the issues in in my spine”  **Tony** “in the career of the army, you know, you think are you gonna get discharged? Are you gonna get MD’d? So yeah, there's always that on the back burner, you know. Can I carry on? What's gonna happen?”  **Tony** “your pain’s there for a reason. It's your brain, or your body saying to your brain, ‘oh, there’s a little something here and something here’, you know. Like your car bringing up a light on the dashboard, you know, it's still gonna work, but there’s a light on the dashboard saying there’s a problem, and if you don't fix that problem or look at that problem in the short term, then in the long term it's gonna cause more problems. So, you know, it's like engine lights on, and if you leave it on a couple years later your engine will blow up because you haven't addressed the actual problem”  **Catherine** “I have had two x rays of my hip so, and it was, I was horrified because the first one was like in my early 50s so I had a evidence [of osteoarthritis] when I gave up high impact stuff. But since it, and I've always wondered at the back of mind, if I'm making it worse by, because I do a lot of walking”  **Bethany** “I think it's just getting worse. And then, obviously, as you get older all the things start to break down a bit, and I've just got crap bones. So, as I keep saying, I’ve been given the crap bones”  **Bethany** “So last year, or the year before, I bought some roller skates because I used to skate all the time as a teenager. And I've been on them a few times, but I'm really conscious that if I fall I'm going to break something and hurt myself even more”  **Hannah** [on exercise leading to CMP] “I mean looking back on it now, the slipped discs or the bulging discs just didn't happen overnight. I must have been making them worse over a series of years, so I definitely think, not being a natural runner anyway, I took up running quite late, I was in my late thirties, I think, yeah, I definitely think cycling, will have, I don't think it caused it, but it will have definitely contributed [to CMP]” |
|  | Avoidance | **Catherine** “Now I would always use a rucksack [instead of shoulder bag]. And with my hands I have I stopped, I had to stop doing those things because it is just too sore. I've got an upright mouse for work, and I've got dragon software for typing, so I should reduce my typing. I've got a cleaner for cleaning. And my, sorry my back, I only rarely wear heels now, and I only wear them couple of times a month. So I'm just really aware that flat shoes are much better, and they probably also help my sacroiliac and leg pain as well” **MD** “Now, stopping any of those things; do you think the fact that you stopped them, or reduced them, or modified them, could have actually made pain worse?” **Catherine** “The things that we've mentioned? No, no, it helped. They all helped”  **Bethany** “My husband has been amazing. He'll do anything for me, and like when I've had a bad flare up he’ll go up and down, even though he's working still and fetching [daughter] from school. He is amazing”  **Bethany** “That's when I started getting the help from the occupational health and putting like rails in the house and stuff because I would struggle to like, lift myself up that in the bath, or like, when it was really bad, put my leg over the bath so I ended up with like a bath seat so I could like slide on to it”  **Hannah** “So as soon as I discovered that I got slip discs in 2016, I stopped doing some activities, I stopped riding my bike, I used to run a bit, I was never a natural runner, but I stopped doing that”  **Tony** “I'm not mobile at the minute. If I need to go anywhere I got the wheelchair if I need to. You know. I got sticks”  **Bethany** “I mean I've slowed the process down by changing my career, by helping myself more when I feel my body is going so I can give it a rest”  **Bethany** “when I get in, as soon as I get in from work, I get in bed for an hour, because I am physically and mentally just knackered... just like, oh, stay in bed then”  **Bethany** “That's when I started getting the help from the occupational health and putting like rails in the house and stuff because I would struggle to like, lift myself up that in the bath, or like, when it was really bad, put my leg over the bath so I ended up with like a bath seat so I could like slide on to it”  **Bethany** “When it first started, I did have a really bad bout of depression. So when it was school holidays or weekends, I’d be like to my husband no you take the kids for a walk or whatever and I’m going to go to bed for a couple of hours, so a lot of time spent sleeping, or hiding in the world of somebody else on TV series and stuff”  **Hannah** “in an ideal world, if I could not work at all, that would be the best, because I could just do what I felt I wanted to do that day, or felt what I could do rather than having to do some things that will actually use up some of my energy that I won't then have for later, and then has a knock on effect on the next day” |
|  | External locus of control | **Tony** [on why pain didn’t resolve] “[lack of] treatment, I would say. When I say treatment, I mean, doesn't mean medication, it could be a certain type of therapy. So your back pain, let's see what we can do. Is it going to be injection, is it going to be this. Yeah. They sort of, I've had a thing where it’s “oh, you can’t have an injection because it's not good for you, you can't have this, you can’t have this””  **Tony** [on why pain didn’t resolve] “I'd say lack of fully investigative work, you know. Yeah. If it had been looked at the start exactly, and gone in more in depth, then maybe it could have lessened it”  **Tony** [on why pain didn’t resolve] “So maybe if I had a person-centred mot, as it would be, to start off with. The diagnosis, looking into it, and actually not just ‘here’s some medication, off you go’, you know. Let’s look it a little bit deeper, you know. ‘What else can we do? How can we alleviate this so it can go away’, or it can be, you know, a lot better than it is, so it doesn't degenerate as quick as it has done”  **Bethany** [on what could have helped] “Probably going to the doctor sooner. Maybe they could have helped with the degeneration, they could have, or even now they could assist me a bit better with more knowledge on it”  **Bethany** [on seeking pain medication from the GP] “Well, “why do you need it?” it's like, “because I'm having a flare up”, “but why don't you try this instead?”, it's like “because that doesn't work for me”” |
| **Positive coping strategies contribute to CMP** | Solution focussed coping | **Hannah** “the GP said to me “I think you've got fibromyalgia”. I mean it's never been a formal diagnosis; for me, I don't really need one. I'm happy to work with that and manage it from what I've read about it since. It seems to make sense to me. But having something that I can then go research and work out how to work with helped me, because I like to know about things”  **Hannah** “So it's a bit of like learning about it, then learning about me. But as you just indicated then I was like ‘okay, well, so what do I do about this?’, you know, and ‘what do I do to manage myself better?”  **Hannah** “I mean, this is my view of fibromyalgia, it lies with me. It lies with the individual. It lies with me to decide how I'm going to let it affect me or not affect me”  **Charlotte** “I’ve always, because I've always been reasonably active, and it's only occasionally been acute. I've just sort of carried on”  **Edward** “I think a positive attitude is the most important thing; not saying ‘oh, dear, I shall never walk again’ which presumably some people do say” |
|  | Positive attitudes | **Edward** [on if his psychological experiences had made CMP worse] “No, I don't think so. Quite the opposite if anything. I think these are fairly positive thoughts, positive meaning ‘get over it, kid’ sort of thing, you know”.  **Charlotte** “Oh, sometimes I get fed up with it. You know. It would be nice if it wasn't there. But no, I just try and get on”.  **Hannah** “I think I'm quite a self-motivated person, and I will go and find things out, and I will read things, and I will make my own decisions. But I think for a lot of people they aren't necessarily self-starting like that”.  **Edward** “I think a positive attitude is the most important thing; not saying ‘oh, dear, I shall never walk again’ which presumably some people do say”.  **Edward** “I'm a basically a fairly optimistic person or a fairly determined person to get on with my life as well as possible. Probably always have been like that. I'm fairly intelligent, so I can think round, implications, and applications”.  **Edward** “Going into a hospital, probably, that event probably did frighten me a bit, but it didn't frighten me in the sense of ‘my God, this is the end of my life’, or ‘I've been doing something totally wrong and I must change everything’. My main concern was to get back to work as quickly as possible, which I suppose again, is a positive thought rather than a negative one”. |
|  | Exercise | **Edward** “Some people say you should rest but I don't believe that. I think you have to continue to use ligaments or joints that are not working properly”.  **Edward** “So, when I get twinges when I'm walking, I just keep walking. When I get pains in my wrist, depends what I'm doing, I might sometimes try to do this if this wrist gets particularly painful, or we've got those squeezy ball things, I haven’t got one on this desk, I think, you know you can exercise with”.  **Tony** “Yes, I would say, yes… if you’re active, regardless of what that activity is, anything is good for the mind and the body”.  **Charlotte** [about the impact of Covid] “I couldn't go swimming. And that was a major problem. And I, I had to, my exercise had to be walking. And I found that quite difficult… It’s much better when I’m able to keep active. If I'm not active, I would seize up”.  **Catherine** “if I don’t do exercise my back pain is worse”.  **Hannah** “So, I started Yoga in 2016, once I'd seen the consultant and was happy that that would be okay for me to do. And I carried on with swimming”. |
| **Unsatisfactory healthcare contributes to CMP** | Let down by healthcare services | **Bethany** “I think they left me because they didn't want to do anything. And I think that, with a lot of pain issues, doctors sort of push you off, and they don't care. It's like you've got to prove that you're in this pain constantly, that you've got to somehow show them their job… And I feel let down, if I'm being honest. That I've just been thrown in the sea to survive. ‘I'll throw you a raft every now and then, but it's got a bit of a puncture, so you have to keep asking us for some more air’. So that when I have a big flare up, it's like trying to get crack cocaine off the Queen or something [getting painkillers from the GP]”.  **Tony** “It's like, yeah, if I’d have had say any, a vast investigation of my back or my spine at that point when I was having all them problems, maybe they could say, ‘oh, look, this is happening here, and here, and here, let's do this, this and this, and that should hopefully alleviate it going on’. Because I never had that”.  **Tony** “I think, If it had more investigation work, even when now I've been to somewhere when I've had a fall and I need to go get my ankle checked out, they won't look at it. They won’t X-ray it. It's like, if they know it's an old injury that hasn't got better then [they’re] not interested in it”. |
|  | Fobbed off | **Tony** “And I’ve fallen straight on my face because it's gone, excruciating pain, flat on your face, you know. Yeah, so it's not good on that side. And I've tried to get help for it loads of times, but because it's an old injury they won’t look at it or do anything to it. So yeah, it's live with it”.  **Bethany** “there was something missing in me, and it was like mourning my old life. The old Bethany. But they don't teach you that in these courses, and I just felt like it was just, just a way to get rid of you”. |
|  | Lack of empathy | **Tony** “there's sometimes people didn't have empathy when I was going through it, and they didn't believe what you had, and it was a struggle to get that help. Like, you know, I want a referral to see a specialist and the doctors say ‘no you can't have it’”.  **Bethany** “And I understand that they just haven’t got the time, and they've got too many patients, and what not. But for the patients that are really struggling, not only physically but mentally, because of the physical issues, they just feel alone. They just feel unheard. It's like you’re talking to a brick wall because nobody's listening because all they'll hear is ‘you're in pain, so you want opioids’, no, I want somebody to help me”. |
|  | Lack of trust in healthcare professionals | **Bethany** “And this is why you get crap doctors and crap carers and stuff like that, because, they don't understand, and they’re there for money, if anything. You need to put your heart in a job before you look at the wages”.  **Bethany** “you live with it, because the doctors say that they can do anything, but they're not willing to help in any way apart from keeping sending you to pain management clinic, and do these little things about CBT, and learning to live with your illness”.  **Tony** “It's like you were fighting, fighting, fighting all the time to get the right diagnosis, the right equipment, the right medication. Yeah, you'd been lied to. So all that, I think, does take a toll as well on the mental side”. |
|  | More peer support options would help | **Hannah** “So, I think if my GP had said to me ‘I think you have fibromyalgia. Here's a support group you can go and talk to locally’, or, you know, ‘here's someone who is a coach and can help coach you through, you know, what you're experiencing’. I think that would have been helpful”.  **Bethany** “It helps talking to other people in the same boat with chronic illness, like on Facebook, living with chronic pain. But the times when you’re really down and things aren't looking too great. You don't want to put upon them people, even though you know that they feel the same way, because it is depressing”.  **Bethany** “How can you tell somebody how to do something when you’ve never lived through it yourself” **MD** “So, with that point… do you think that having people who have been through, or are going through, something similar, you know, so you think that would be helpful?” **Bethany** “Yeah. And I think that since I’ve done my course [as a counsellor]… so I’ve have had quite a lot of things that I’ve had to deal with. So, like I’ve been in a domestic violence relationship years back, and I’ve had a lot of bereavement in the family, and a lot of child abuse when I was younger… you could be the most academic person, and pass with high colours, and still not have the empathy or the life experience [to be able to help]”. |
| **Historical activities contribute to CMP** | Work | **Charlotte** [about why developed CMP] “I put it down, and this is my interpretation, to the fact that I was a teacher and I carried books, and if you're a science teacher you will know that it had masses of books. And I also had two children who I carried on my hip”.  **Tony** “The army was, that's where I got a lot of the problems, I believe, especially the back pain. I was 10 stone, 6 foot 2, and you got a 40kg bergen on your back”.  **Tony** “you’re always trying to do 120 percent… so you didn’t get down from a tank, you jump down from a tank… so yeah, all that, yeah, has gone to the back pain. And now I got my MRI was, yeah, the L2, 3, 4, 5, and S1 bulging”.  **Hannah** [on the cause of slipped discs] “I think a lot of it was down to stress and poor posture and sitting at little tables meant for small people”.  **Bethany** “I used to be a carer in the community. So obviously that was a very physical job lifting old people and whatnot and caring for them. And I just couldn't do it anymore. So that drained my body”. |
|  | Physical activities | **Catherine** “You know, rucksacks weren’t in fashion when I was at school, and I had really heavy bags, and I carried a bag over my left shoulder. So I've always thought that that had something to do with my sacroiliac pain”.  **Hannah** “I put it [CMP] down to playing a lot of sport when I was a teenager, I was outside a lot just putting my body through a lot of physical stuff”.  **Edward** “I assume it's abnormal gait, or something, that makes these things happen”. |
