# Supplementary material for: Why do you think you still have pain? Individuals’ beliefs on the biopsychosocial factors that contribute to their chronic musculoskeletal pain: a qualitative exploration
Source: BMC Musculoskelet Disord. 2025 Dec 24;26:1103. doi: 10.1186/s12891-025-09243-1 (PMC12729344; doi:10.1186/s12891-025-09243-1)
Supplement: Supplementary file 4 — Supplementary Material 4. [file 12891_2025_9243_MOESM4_ESM.docx]

**CODING TREES**

**Biological factors**

**Social factors**

**Psychological Factors**


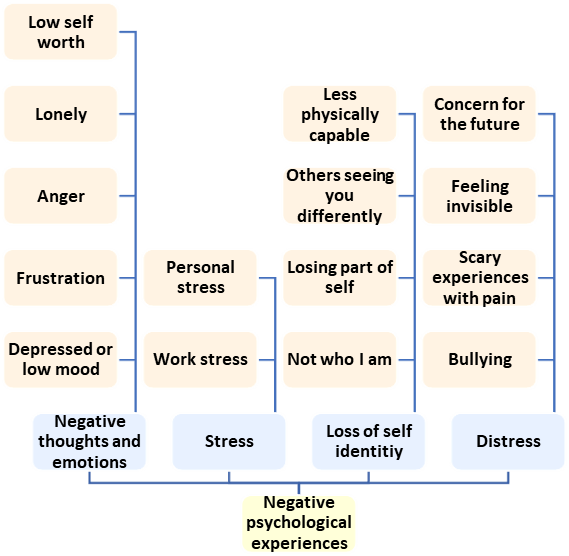

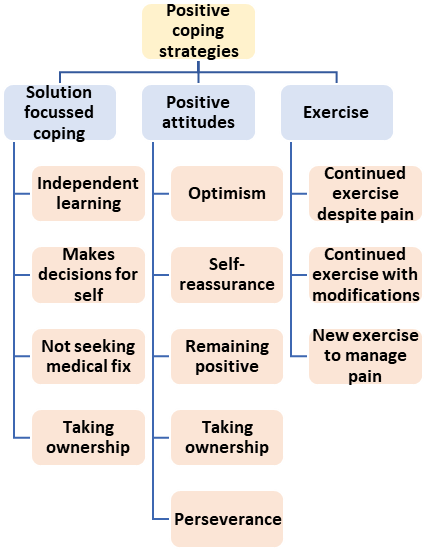

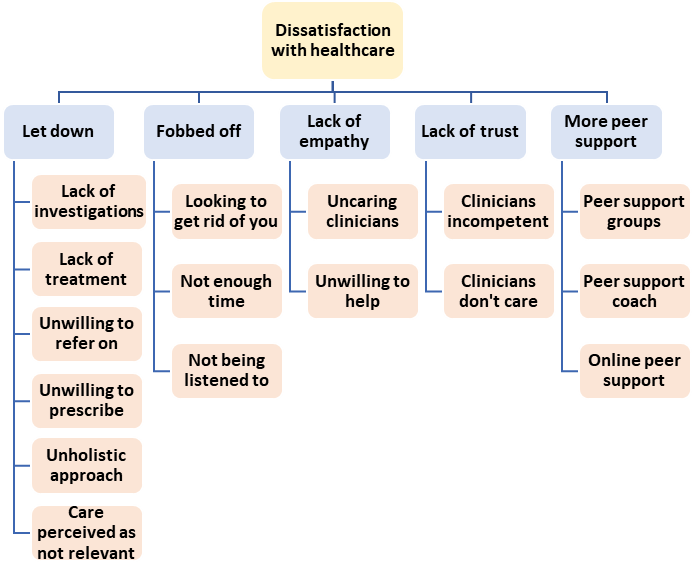

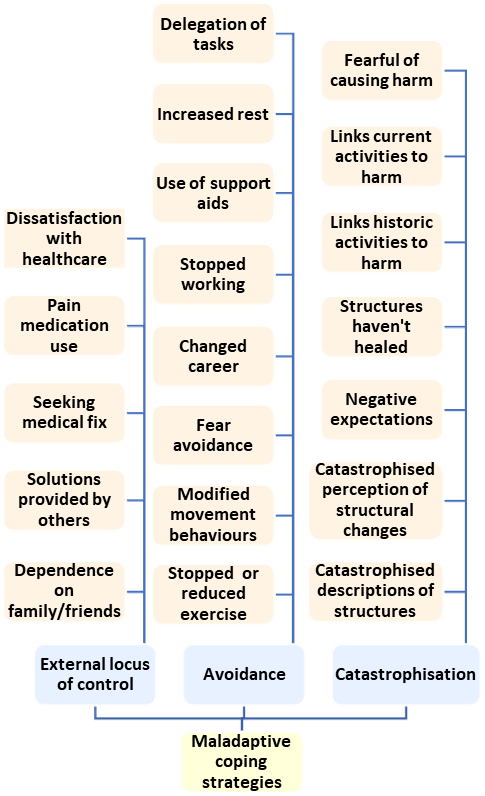


**Psychological factors**
